# Lessons from Nature: Understanding Immunity to HCV to Guide Vaccine Design

**DOI:** 10.1371/journal.ppat.1005632

**Published:** 2016-06-30

**Authors:** Zachary T. Freeman, Andrea L. Cox

**Affiliations:** Division of Infectious Diseases, The Johns Hopkins University School of Medicine, Baltimore, Maryland, United States of America; University of Kentucky, UNITED STATES

Hepatitis C virus (HCV) is an important global health concern with approximately 185 million people infected [1]. HCV infection most often leads to chronic infection with few early symptoms, but chronically infected individuals can develop liver cirrhosis and hepatocellular carcinoma. Genome-wide association studies in humans have identified innate associated genes and HLA class II as important predictors of spontaneous clearance of HCV [2,3], but the correlates of protective immunity are not fully defined. The existence of few models to study protective immunity has hindered vaccine development research. Despite this limitation, significant advancements have been made in our understanding of protective immune responses to HCV using the chimpanzee model and humans exposed to HCV (Fig 1).

**Fig 1:**
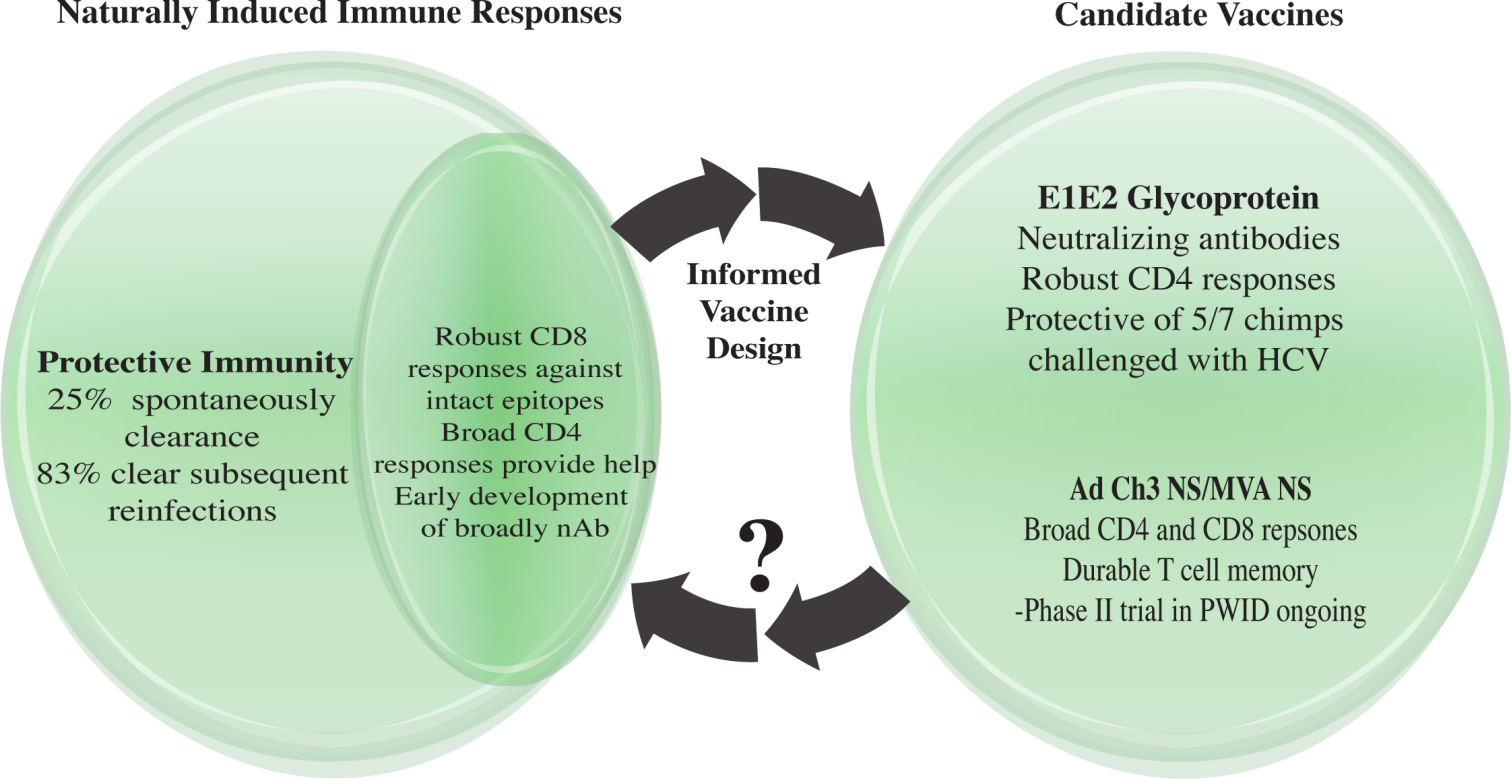
Hepatitis C virus vaccine development has been hindered by few representative models and the recent significant limitation in use of chimpanzees as a model. Although it is not completely understood what confers protection, research in HCV-infected humans who control infection has enhanced understanding of the immune response and informed vaccine design. Two candidate vaccines have been tested in humans. The E1E2 glycoprotein and Chimp Adenovirus 3 Nonstructural (Ad Ch3 NS)/Modified Vaccinia Ankara NS (MVA NS) vaccines have elicited robust immune responses in healthy humans that resemble immune correlates of clearance. The Ad Ch3 NS/MVA NS vaccine is in a phase II clinical trial in people who inject drugs (PWID) to determine if it confers protective immunity. Immune responses identified in those protected from HCV in vaccine trials can be assessed in natural infection as well to determine if they mediate disease control.

## Why Do We Need a Vaccine with the Recent Development of Directly Acting Antiviral Agents?

While recently developed direct-acting antiviral agents (DAAs) cure HCV at high rates, several factors may limit their overall impact. Infections are often difficult to identify because acute and chronic HCV infection are largely asymptomatic [4], and few endemic areas have well-developed screening programs. Even in the United States, only 50% of people who are infected are aware of their HCV-positive status [5,6], and many of the most at-risk populations, such as people who inject drugs (PWID), infrequently seek medical care. A separate concern is that while DAA treatment is effective, it comes at a very high cost. DAAs effectively cure people with HCV infection, but do not protect against reinfection or reverse all complications of liver disease. Given these limitations, a prophylactic vaccine is needed to dramatically reduce the rate of new HCV infections [7].

## What Evidence Exists from Natural Infection Suggesting Protective Immunity?

HCV leads to chronic infection in 75% of people, while 25% of people are able to spontaneously clear the virus. Spontaneous clearance of infection by humans and by chimpanzees does not seem to be sufficient to provide sterilizing immunity against future infections, because reinfections have been documented in both humans and chimpanzees [8–16]. Despite this lack of sterilizing immunity, chimpanzees and humans that clear initial infection often clear subsequent infections [10,14–17]. Humans who have previously cleared a primary infection go on to resolve subsequent infections 83% of the time [14]. Moreover, these individuals have decreased viral titers and more rapid clearance of virus compared to initial infection, suggesting a protective adaptive immune component [14]. Similarly, chimpanzees that spontaneously clear initial HCV challenge are protected from both homologous and heterologous viral challenge with decrease in magnitude and duration of viremia [18]. Reinfection in humans was also associated with broadened cellular response to HCV and an increase in broadly HCV-neutralizing antibodies [14]. Taken together, these data support the role of the adaptive immune system in providing protective immune responses to subsequent HCV challenge, and development of a vaccine to elicit similar adaptive responses may confer protective immunity.

## What Are the Cellular Components of Protective Immunity to HCV?

T cells are an important component of the adaptive immune response to HCV. Considerable data exist from human and chimpanzee studies supporting the importance of HCV-specific CD4+ T helper and CD8+ cytotoxic T cells in clearance of primary infections and reinfections. Chronic infection is characterized by the progressive loss of functional HCV-specific T cells, which leads to inability to clear the virus. Conversely, a strong and broadly directed CD4+ T cell response to HCV has been associated with spontaneous clearance of infection [19–22]. Broadly directed CD4+ T cells may be present during initial HCV regardless of infection outcome, but CD4+ T cells become defective early in those that progress to chronic infection [19]. CD4+ T cells are thought to be important in providing “help” to effector CD8+ T cell responses and generating T cell memory. During acute infection of PWID, selection of mutants that evade CD8+ T cell responses is found in those who progress to chronic infection, but not in spontaneous clearers [23]. Furthermore, antibody-mediated depletion of CD8+ T cells prior to reinfection of chimpanzees that had previously cleared HCV infection led to prolonged viremia that resolved with the reappearance of CD8+ T cells, demonstrating the crucial role for CD8+ T cells in HCV control [13]. The role of CD4+ T cells in HCV control was less clear in chimpanzees because antibody-mediated depletion of CD4+ T cells led to chronic viremia despite the presence of functional intrahepatic CD8 + T cells, and reappearance of CD4+ T cells did not lead to viral control [24]. What generates T cell responses that control HCV infection is unknown, but some environmental cues have been demonstrated to be important. Th17 cells produce cytokine IL-21 and contribute to the maintenance of memory CD8 T cell and antibody responses to HCV [25,26].

## What Protective Effect do Humoral Responses Provide?

It remains unclear if antibodies produced in acute HCV infection affect progression to chronic infection. Patients with hypogammaglobulinemia have been demonstrated to clear HCV despite a lack of antibodies [27]. In addition, a randomized clinical trial testing the administration of human Hepatitis C antibody-enriched immune globulin product (HCIG) to HCV-infected patients undergoing liver transplantation demonstrated that the HCIG had no ability to protect against reinfection of the new liver [28]. While these two studies suggest that antibodies are neither crucial nor sufficient for HCV protection or clearance, recent work with neutralizing antibodies (nAbs) has provided evidence of antibody-mediated immunity to HCV. Evidence in humans of protection from nAbs initially came from two common source infections. In the first of these outbreaks, patients with the highest level of nAbs in the serum also had the lowest level of viremia [29]. In the second outbreak, early development of nAbs correlated with clearance of virus, while later development of nAbs was associated with chronic infection [30]. Early generation of broadly nAbs has also been associated with spontaneous clearance in PWID [31]. Work in mouse models containing human liver tissue has demonstrated that broadly nAbs can prevent HCV infection when administerered prior to or during infeciton [32]. While broadly nAbs appear to be important in spontaneous resolution of HCV, little is known about the epitopes of HCV required to generate these responses, and the infecting HCV genotype seems not to dictate how broadly nAb responses are generated. In one study, some subjects infected with genotpyes 1,2, or 3 were able to mount broadly nAb responses against a genotype 1 pseudoparticle library [31].

## Where Are Current Efforts at Developing an HCV Vaccine?

HCV is characterized by enormous sequence diversity exceeding that of HIV [33]. The large number of HCV strains and high degree of diversity even within a single strain represent significant barriers to the development of an effective vaccine. Several strategies have been attempted in mouse models and chimpanzees to develop protective vaccines against HCV, but few have moved to chimpanzee or human trials. The difficulty of assembling at-risk cohorts and the high cost associated with administering these trials remain major limitations in testing new HCV vaccines. Chiron (now Novartis) developed a recombinant envelope vaccine containing E1E2 glycoprotein with the MF59 adjuvant that induced neutralizing antibody responses in chimpanzees [34]. In these animals, high antibody titers at the time of challenge protected from infection after viral challenge [35]. Two of seven animals had low antibody titers at the time of viral challenge, and both developed chronic infection. This vaccine also induced neutralizing antibody production and robust CD4+ T cells in healthy human volunteers, but no further progress on this vaccine strategy has been reported. A second vaccine, the Chimp Adenovirus 3 Nonstructural (Ad Ch3 NS)/Modified Vaccinia Ankara NS (MVA NS) vaccine, has been developed that elicits broad and strong T cell responses. This vaccine consists of a chimpanzee adenovirus 3 construct vector containing the relatively conserved nonstructural proteins of HCV for the priming vaccination followed by modified vaccinia Ankara (MVA) expressing the same nonstructural proteins as the boost vaccination. Challenge of healthy people not at risk for HCV infection elicited broad CD8+ T cell responses characterized by robust production of interferon-γ, memory cell development, and durable maintenance of HCV-specific T cells [36]. A phase II clinical trial of this vaccine is underway in a cohort of PWID (clinicaltrials.gov, NCT01436357), and it remains to be seen if the immunity generated is protective against persistent HCV infection.

Spontaneous clearance of HCV by both humans and chimpanzees has yielded important evidence of protective adaptive immunity to HCV. CD4+ and CD8+ T cell responses are key determinants of protective immunity, but broadly neutralizing antibody responses likely contribute to protection. The successful generation of broad immune responses by recombinant envelope and T cell vaccines confirms the ability to generate HCV-specific neutralizing antibody and CD4+ and CD8+ T cell responses. This T cell vaccine does not aim to create sterilizing immunity, but instead aims to control infection as observed in humans who become repeatedly viremic without developing chronic infection. It remains to be seen if these responses will be sufficient to prevent development of chronic infection in at-risk cohorts. Existing data from spontaneous clearance of HCV suggest that guided design of future vaccines should target development of broad CD4+ and CD8+ T cell responses as well as generation of broadly neutralizing antibody responses (Fig 1). Vaccine trials will likely also provide enhanced understanding of the correlates of protective immunity.
